# Assessment of lead exposure in indoor shooters in central Poland

**DOI:** 10.1038/s41598-023-39847-3

**Published:** 2023-08-03

**Authors:** Adam Darago, Michał Klimczak, Joanna Jurewicz, Małgorzata Kucharska, Anna Kilanowicz

**Affiliations:** 1 Department of Toxicology, Medical University of Lodz, Muszyńskiego 1, 90-151 Lodz, Poland; 2 Department of Chemical Safety, Nofer Institute of Occupational Medicine, Św. Teresy 8, 91-348 Lodz, Poland; 3 Central Laboratory, Nofer Institute of Occupational Medicine, Św. Teresy 8, 91-348 Lodz, Poland

**Keywords:** Occupational health, Risk factors

## Abstract

A steady increase in shooting practices is observed worldwide. Potential lead exposure at shooting ranges poses a risk to their employees and users, which is not widely reported outside of the USA, especially in Poland. Exposure to lead results from the use of bullets containing lead and the main route of exposure to this metal at shooting ranges is inhalation, i.e., during shooting or cleaning. The aim of this study was to assess lead exposure of employees and users in selected indoor shooting ranges in central Poland. Airborne lead concentrations at all locations in the shooting ranges were above Polish occupational exposure limit (OEL, 0.05 mg m^−3^). Elevated blood and urine lead levels, and decreased 4-aminolevulinic acid dehydratase activity (ALA-D) were found in subjects participating in shooting even for only a few (< 10) hours per week. Lead exposure at shooting ranges in central Poland, as indicated by elevated blood lead levels and decreased ALA-D activity, could represent an elevated risk for adverse health effects. Thus, information on the possible health consequences of lead exposure should be provided at these sites, and biomonitoring appears to be reasonable for regular workers and shooters.

## Introduction

Recent years have seen a steady increase in interest in shooting and, consequently, in the number of people potentially exposed to lead at indoor shooting ranges. In the United States alone, there are approximately 16,000–18,000 indoor shooting ranges that employ tens of thousands of workers^1^. Interest in shooting and shooting sports is also gradually increasing in Poland and other EU countries. It is estimated that there are about 80,000 open and closed shooting ranges in Europe, with more than 460 being in Poland^2^.

While in various industries (e.g., battery or pigment production, and printing) the problem of occupational exposure to lead is very widely described and regulated by law, no such information or formal regulations exist regarding the safety of employees or users at shooting ranges in Europe. It is well known that occupational exposure to lead is particularly critical in the hematopoietic system, the cardiovascular system, the peripheral nervous system and the kidneys^3^.

Exposure to lead in indoor ranges results from the use of bullets, most of which contain lead. The metal is present in large quantities in the primer (as lead azide or lead styphnate), which burns in the barrel of the firearm to provide propulsion for the projectile^4,5^. Lead is, therefore, present in the air of shooting ranges in the form of particles, dust and vapours, and the main route of exposure to lead at shooting ranges is inhalation, i.e., during shooting or cleaning^3,6,7^. It has been shown that during shooting in the indoor shooting ranges lead is concentrated in particles in the range of 100 to 2000 nm. Particles of this size are easily and almost completely (up to 95%) absorbed when inhaled^8–10^, which increase the risk of toxic effect of lead.

An additional source of exposure is inadvertent ingestion of lead-containing particles deposited on surfaces, which may be transferred with hands or clothing (the dermal absorption of lead is negligible)^3,6,7^. Unfortunately, it is still common for both instructors and users to take a meal right after shooting, without washing their hands first, and not to use protective clothing when operating shooting ranges, which significantly increases the risk of ingestion. Thus, ensuring safety at shooting ranges due to exposure to lead compounds is crucial^11^.

According to the requirements of Council Directive 98/24/EC of 7 April 1998 on the protection of the health and safety of workers from risks related to chemical agents at work, blood lead level (BLL) determinations are mandatory in persons occupationally exposed to lead compounds. The maximum BLL in European Union (EU) was set at 700 µg L^−1^, but medical care should be provided to workers with values above 400 µg L^−1^. In addition, other biomarkers are recommended in the assessment of lead exposure, such as 4-aminolevulinic acid dehydratase (ALA-D) activity, urinary 4-aminolevulinic acid (ALA) concentration or zinc protoporphyrin (ZPP) concentration. The EU OEL (occupational exposure level) of lead and its inorganic compounds in work environment air is 0.15 mg m^−3^^12^, but could be lower in individual EU members states (range in UE member states: 0.004–0.15 mg m^−3^^13^; in Poland: 0.05 mg m^−3^^14^).

A review of the literature indicates that the number of chronic lead poisoning cases in shooting range workers has increased in recent years. These individuals were found to demonstrate BLLs ranging from 430 to 820 µg L^−1^ and symptoms of poisoning such as paraesthesia, increased blood pressure, abdominal cramps and fatigue^7,15,16^.

Although the problem of lead exposure at shooting ranges is extensively described in the USA, much fewer data are available for Europe, and none, for Poland, where many indoor shooting ranges are set inside buildings previously used for, among others, industry or agriculture. Hence, the aim of this study was to assess the lead exposure of employees and users in selected indoor shooting ranges in central Poland on the basis of biomarkers of exposure, i.e., BLLs, urine lead levels (ULLs) and ALA-D activity in the blood, as well as lead concentrations in the air at shooting ranges.

## Results

### Airborne lead concentrations

**Figure 1:**
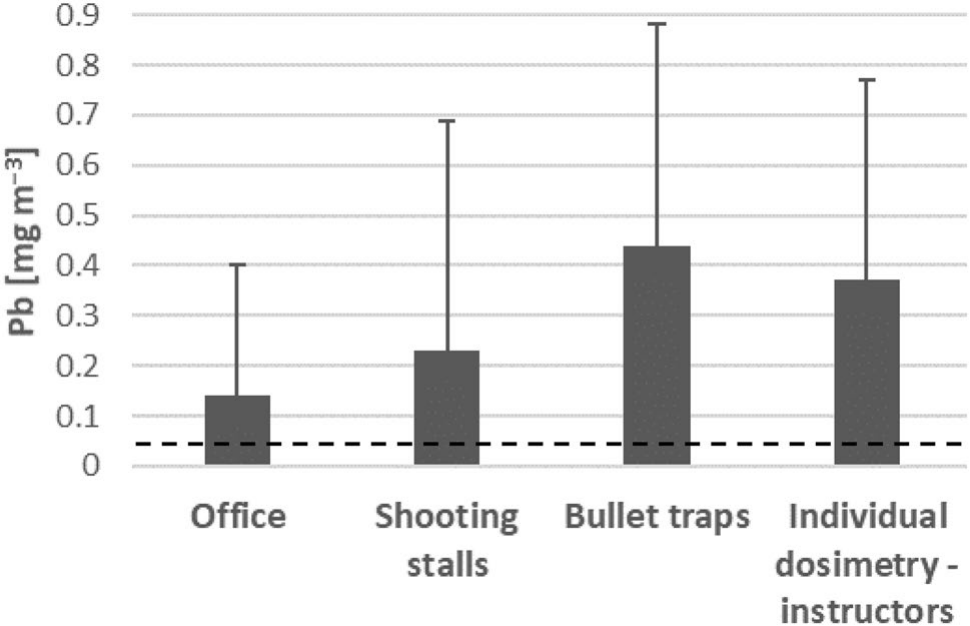
Concentrations of lead (Pb) in the air of shooting ranges. Data are presented as mean + SD. Dashed line represents Polish OEL (0.05 mg m^−3^).

Figure 1 shows the lead concentrations identified at various locations of the shooting range (stationary samples) and based on individual dosimetry. In the air samples, lead concentrations ranged from 0.01 to 0.70 mg m^−3^. The lowest concentrations (0.140 ± 0.013 mg m^−3^) were found in the offices located within the shooting ranges, while the highest concentrations (0.44 ± 0.22 mg m^−3^) were found at the bullet traps. The sand berm-type bullet traps demonstrated higher levels of lead than the steel-rubber ones. The mean lead concentration in the breathing area of instructors (measured using individual dosimetry) was in the range of 0.37 ± 0.20 mg m^−3^, which was higher than the values measured at shooting stalls (0.23 ± 0.23 mg m^−3^). This difference may result from the need for instructors to change targets at the bullet traps, as none of the shooting ranges were equipped with an automatic target changing system. Lead concentration in the air at all locations exceeded the Polish OEL for lead and its compounds, that is, 0.05 mg m^−3^^14^.

### Biomarkers of lead exposure

Table 1 shows the results of BLLs, ULLs and ALA-D activity in the study groups. BLL values were significantly higher in all subgroups than the values in the control group and reference values for the general population (below 50 µg L^−1^ in Poland^14^). BLLs in the study group increased with the time spent at the shooting ranges. It was noted that the BLL values in subgroup C (> 20 h week^−1^) were twice as high as those in subgroup A (< 10 h week^−1^) and around 1.5 times higher than in subgroup B (10–20 h week^−1^).

**Table 1:** Concentration of lead in blood (BLL) and urine (ULL) and ALA-D activity in study groups in relation to time spent at the shooting ranges.

		Control group (43)	Study group		
			< 10 h per week (17)	11–20 h per week (13)	> 20 h per week (15)
Exposure	[years]	0	6.1 ± 7.2	6.4 ± 5.4	12.9 ± 11.1
	[h/week]	0	6.1 ± 2.6	17.4 ± 3.5	35.7 ± 5.6
Blood lead level (BLL) [µg L^−1^]		11.79 ± 7.19	114.62 ± 48.64^a^	174.80 ± 33.93^a^	281.42 ± 62.26^ab^
Urine lead level (ULL) [µg g^−1^ Creatinine]		0.35 ± 0.26	6.13 ± 5.32^a^	29.51 ± 20.40^a^	51.09 ± 18.75^ab^
ALA-D activity [µmol min^−1^ L^−1^]		63.17 ± 5.87	29.15 ± 8.80^a^	23.89 ± 10.58^ab^	9.54 ± 1.42^abc^

^a^Statistically significant compared with control (*p* < 0.05).

^b^Statistically significant compared with subgroup A (*p* < 0.05).

^c^Statistically significant compared with subgroup B (*p* < 0.05).

Another parameter studied was the activity of ALA-D, which significantly decreased, almost threefold, in subjects in the study group (21 µmol min^−1^ L^−1^) compared with controls (63 µmol min^−1^ L^−1^) (Table 1). The exposure time had a clear influence on the inhibition of ALA-D activity. Significant inhibition of this enzyme, i.e., around 50%, was even noted in subgroup A, that is, in persons attending shooting ranges less than 10 h per week. The greatest inhibition was noted in subgroup C and was around 85% of control values; this change was significant. This subgroup also demonstrated considerably lower ALA-D activity than the other two subgroups.

**Figure 2:**
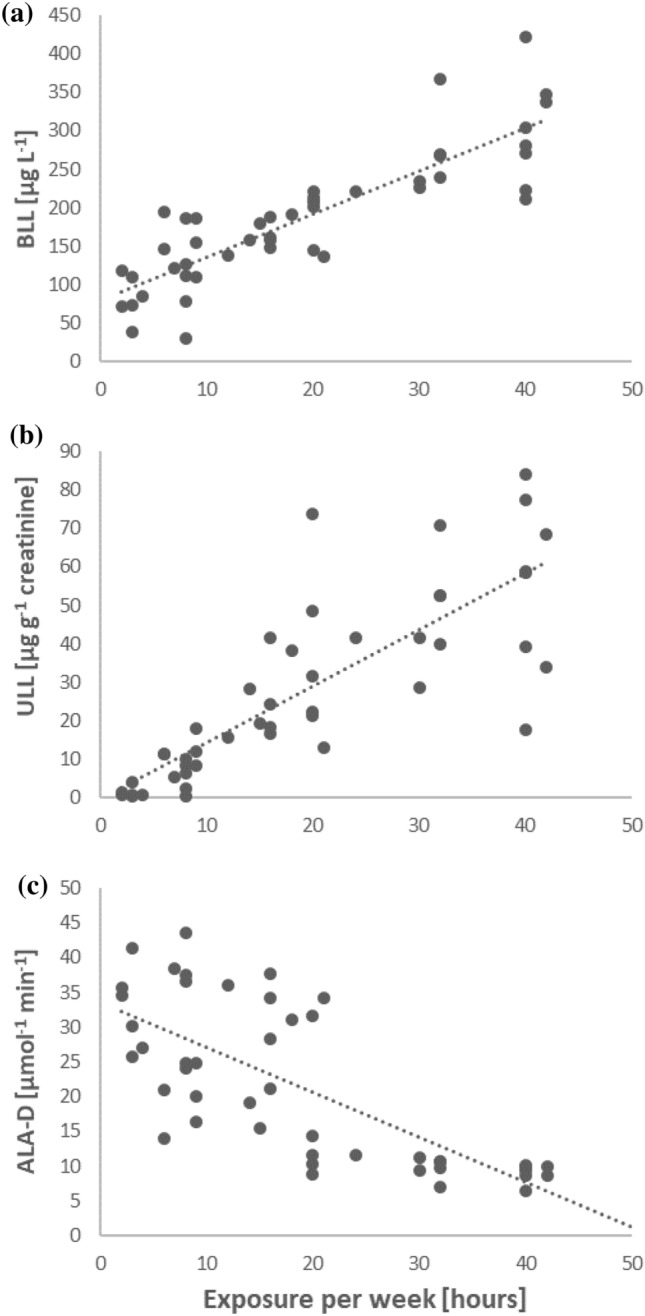
Relationships between time spent at shooting ranges and: (**a**) BLL; (**b**) ULL and (**c**) ALA-D.

A moderate correlation (r = 0.44) was found between BLL and duration of work (years) at the shooting ranges (data not shown). In addition, a very strong correlation (r = 0.79) was found between BLL and the mean time spent at the shooting range per week (Fig. 2a).

**Figure 3:**
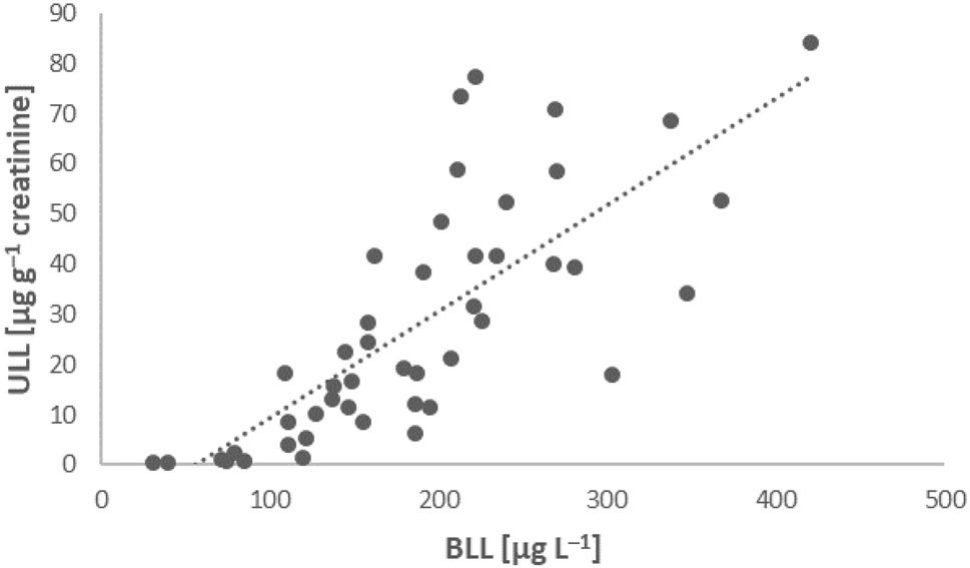
Relationship between BLL and ULL in subjects from the study group.

ULLs in the study group were nearly 100 times higher than those in the control group, and these values increased with the time spent at the shooting ranges. A significant correlation (r = 0.88) was found between BLL and ULL in all subjects in study group (Fig. 3) as well as in control group (r = 0.81, data not shown).

A significant correlation (r = 0.87) was noted between ULL and the time spent at the shooting ranges in the study group (Fig. 2b). However, further analysis showed that as the time spent at the shooting range increased, the correlation coefficient significantly weakened from r = 0.65 (Subgroup A) to r = 0.16 (Subgroup C; Fig. S2 in Supplementary information). The correlation between ULL and duration of work (years) at the shooting ranges was weak (r = 0.37, data not shown).

**Figure 4:**
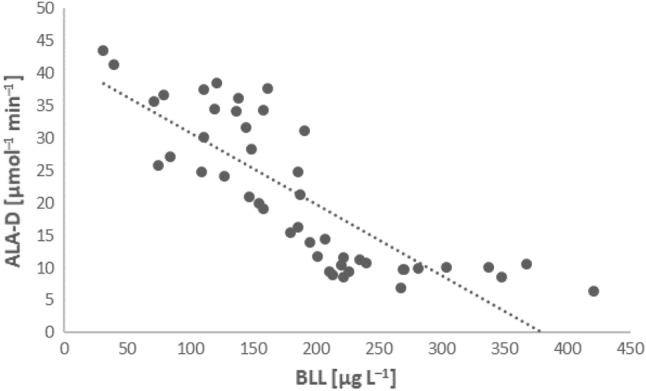
Relationship between ALA-D activity and BLL in the study group.

A very high, negative correlation was found between BLL and ALA-D activity in the study group (r =  − 0.81) (Fig. 4), as well as between ALA-D activity and the time spent at the shooting ranges (r =  − 0.74, Fig. 2c). No significant correlation between BLL and ALA-D activity was found in the control group (data not shown).

## Discussion

Beside benzene or dioxins, lead and its compounds are regarded by the WHO as some of the ten most dangerous chemicals affecting human health worldwide^17^. Although sources of lead exposure are well defined and widely described in the literature, exposure associated with shooting ranges is receiving growing attention. It is estimated that this source is the second largest global source of environmental contamination by lead, after the battery industry, and approximately 10,000 to 60,000 tons of lead are annually released at shooting ranges worldwide^18^. The issue of lead exposure at shooting ranges has been most extensively documented in the USA; less attention has been paid in Europe, and no studies have so far been performed in Poland.

Shooting at ranges results in the release of lead particles, dust and vapours, and considerably high airborne lead concentrations (up to several mg m^−1^) have been found at indoor shooting ranges^19,20^. Air monitoring is the main non-invasive method applied in occupational exposure assessment, including exposure to lead. However, the results of this method only indicate external exposure, i.e., the whole dose to which a person is exposed. When inhaled, the absorption of lead particles is influenced by their size and solubility in water. Particles deposited in the alveolar regions are almost completely absorbed. Only the fraction of the whole dose absorbed and distributed throughout the body (internal exposure) may cause various adverse health effects. Blood lead concentration (BLL) is the major biomarker of current exposure to lead, reflecting internal lead exposure. BLL is also clearly associated with external exposure^8,19–22^.

A biological limit value (BLV) for BLL of 150 µg L^−1^ has been proposed by the ECHA for both inorganic and organic lead compounds, and this should protect workers from lead toxicity^21^. A recent review of BLL data papers published between 1975 and 2016 revealed the widespread occurrence of very high BLLs in shooters, including recreational ones. At least one case of BLL above 100 µg L^−1^ in shooters was found in every paper reviewed, with some exceeding 200 µg L^−1^ and even 400 µg L^−1^^19^. For example, in a study in South Africa, the mean BLL in shooters was 128 ± 105 µg L^−1^, with more than 45% of values greater than 100 µg L^−1^ and over 10% of values greater than 200 µg L^−1^^7^. Highly elevated BLLs in shooters were also noted in South Korea, Mexico and the USA^15,19,23^. In our study, the mean BLL in shooters was 187.33 ± 85.37 µg L^−1^, with concentrations exceeding 150 µg L^−1^ in more than 60% of subjects and exceeding 300 µg L^−1^ in 10% of subjects. Given that the derived biological reference values (for the general population) of BLL in adult men and women are 40 and 30 µg L^−1^, respectively^24^, these figures clearly confirm significant exposure to lead at studied shooting ranges worldwide. This may be a significantly problem for recreational shooters, who are not included in any assessment methods of lead exposure.

The duration of stay at shooting ranges is a very important factor for assessing lead exposure. Madrid et al. found significantly higher BLLs in individuals who had more than 12 shooting practice sessions per year (83 ± 24 µg L^−1^) in comparison with those having fewer than 12 (52 ± 25 µg L^−1^)^23^. Even though our present findings do not suggest any association between BLL and years of shooting practice, a significant increase in BLLs was found to be related with the duration of stay at shooting ranges (expressed in h week^–1^). Those spending more than 20 h week^–1^ at the ranges demonstrated twice the BLL of those spending < 10 h per week^−1^ there and about 1.5 times as much as those spending between 10 to 20 h week^−1^ there. The duration of stay at shooting ranges is also associated with the number of fired bullets, as previously noted by Tripathi et al.^25^. Asa-Mäkitaipale et al. noted a positive correlation between BLL in sport shooters and the number of bullets fired in both the previous year and the previous month^26^.

Another biomarker of lead exposure is the activity of 4-aminolevulinate acid dehydrogenase (ALA-D), which is also thought to be the most sensitive indicator. By inhibiting this enzyme, lead blocks the biosynthesis of heme, and thus haemoglobin, which is one mechanism of lead-induced anaemia^27–29^. Thus, ALA-D activity is negatively associated with BBL. Although no BLV value exists for this biomarker, it could be applied in the assessment of lead exposure-induced anaemia or solely lead exposure. Huang et al. (2020), using multiple linear regression, proved that BLL is inversely associated with ALA-D activity. Because ALA-D activity significantly decreased at BBL > 50 µg L^−1^, this value could be a possible threshold for ALA-D activity as an indicator of lead exposure^30^. A strong correlation between these two biomarkers was also observed in our study. Around 50% ALA-D inhibition, compared with controls, was even noticeable among individuals with the lowest BLLs (around 50 µg L^−1^), and ALA-D activity considerably decreased with the duration of stay at the shooting ranges. As such, ALA-D activity appears to be a potentially valuable biomarker of lead exposure with a BLV of 50 µg L^−1^, as proposed by Huang et al.^30^, and could also be used for the assessment of lead exposure at shooting ranges.

The second aim of this study was to assess the lead concentrations in the air at the shooting ranges where the study group members worked and/or trained. It has been known for several decades that shooting at indoor shooting ranges can result in significant exposure to airborne lead from bullets and primers^31–33^. Moreover, many authors have highlighted that exposure to high airborne lead concentrations in indoor ranges could affect the health of shooting instructors^31,34,35^. In order to protect the health of workers exposed to lead, thresholds have been set for its level in the air of workplaces. In 2019, the ECHA proposed an OEL of 0.03 mg m^−3^ for inorganic and organic lead compounds as an 8-h time-weighted average. This value is an “action level” according to OSHA recommendations; when it is exceeded, the employer has to initiate specific safety measures, even if the threshold value has been set to 0.05 mg m^−3^^36,37^.

Many studies have reported very high airborne lead concentrations in indoor shooting ranges significantly exceeding OELs. Wang et al. found airborne lead exposure levels to range from 0.03 to 3 mg m^−3^ at indoor and outdoor shooting ranges^20^. This is confirmed by our present study, in which airborne lead concentrations ranged from 0.01 mg m^−3^, in administration offices, to nearly 0.7 mg m^−3^, near bullet traps. In a study by Park et al., the air concentrations of lead in beaten zones (0.1–23.4 mg m^−3^) were even 100 times higher than those in the waiting room (0.03–0.27 mg m^−3^)^38^. Scott et al. also reported that the highest lead exposure was obtained while handling the bullet traps, with these values being higher than those linked to shooting^39^.

The most probable causes of lead exposure at shooting ranges are the lack of adequate ventilation and/or personal protective equipment. In our present study, the highest concentrations of airborne lead were determined near the bullet traps, and almost all participants admitted not to use personal protective equipment (PPE). The value of PPE use is confirmed by the fact that higher lead concentrations were obtained using personal dosimetry than with stationary measurements, as dosimetry reflects the actual inhalation exposure due to collection of air from the breathing zone. The presence of high concentrations of airborne lead may have resulted from the fact that as most commercial shooting ranges in Poland, the studied ranges were set inside buildings previously used for various types of industrial or agricultural production and lacked sufficient ventilation for shooting ranges. The nature of this problem was demonstrated by Ramsey et al.^40^, in whose study airborne lead concentrations in a shooting range were significantly reduced from 0.34 to 0.009 mg m^−3^ following modernization of ventilation^40,41^. Considering our present findings (0.37 mg m^−3^), it is very likely that inadequate ventilation is the main reason for such high exposure to lead at our studied sites. It is worth mentioning that size mode of particles emitted during indoor shooting is usually up to 2000 nm. This pose a high risk for extensive lead absorption in the lungs, and thus long-term health risk from lead exposure at indoor shooting ranges^9^.

Our study was probably the first to analyse the problem of lead exposure at indoor shooting ranges in central Poland. The basis for the study was the noticeable increase in the numbers of both people shooting and shooting ranges in our country. The results indicate that as in other countries, lead exposure at shooting ranges may affect human health, regardless of the number of hours spent at these places per week. However, our study has some limitations. Due to the not very large study subgroups, the results obtained, although statistically significant, are rather preliminary and confirm the problem of lead exposure at indoor shooting ranges. These results should not be extensively generalized. There is a need for more in-depth research, especially with regard to the associations between airborne lead levels and biomarkers of exposure.

In conclusion, significant changes in the biomarkers of lead exposure were identified in people who worked or regularly attended selected indoor ranges in central Poland. More specifically, elevated BLLs and ULLs were noted in instructors/employees and shooters participating in shooting practice, even for only a few hours per week. These effects result from the high lead concentrations in indoor air, most likely deriving from lead-containing ammunition. As it could not be ruled out that increased BLLs may cause health problems in shooters, we recommend that information on the possible health consequences of lead exposure be provided at shooting ranges. As the highest risk of lead exposure is associated with attendance at indoor shooting ranges for a few hours each week, biomonitoring seems to be reasonable and highly recommended for regular shooters and workers.

## Methods

### Study design—selection of shooting ranges and participants

Four typical indoor shooting ranges in central Poland were chosen as being representative of shooting activity and general range type. The selected ranges have been in operation since 2010–2015, with 40–150 shooters per day. All used escalator (n = 1), steel-rubber plate (n = 2) and sand berm (n = 1) ball trap types, and the approximate area of the sites ranged from 150 to 800 m^2^. The number of lanes varied from 4 (n = 1) to 8 (n = 1) and 10 (n = 2). All ranges used full metal jacket (FMJ) and lead-type pistol and rifle ammunition in calibers ranging from .22LR, as well as shotgun ammunition (e.g., 12/75 mm) and air rifle/pistol ammunition from various suppliers. In addition, users regularly brought their own ammunition, resulting in different types of ammunition with different compositions being used at each range.

The study group comprised a total of 45 people, including shooting instructors (n = 16), maintenance/cleaning staff (n = 5) and sports club members training at the shooting ranges (n = 24). Their age ranged from 18 to 70 years (42.6 ± 11.5) with presumably different levels of lead exposure. The study was approved by the Bioethical Committee of Medical University of Lodz, Poland (resolution RNN/86/22/KE of 10 May 2022), and written informed consent was obtained from all participants. All participants trained several times a week at the ranges. The study group was divided into three subgroups based on the time spent at the shooting range, i.e., < 10 h per week (subgroup A), 11–20 h per week (subgroup B) and > 20 h per week (subgroup C). All persons declared not to use any personal protective equipment (including masks). Only two of the tested cleaning staff confirmed the use of protective clothing and FFP2-/FFP3-class dust masks during work.

A separate control group was also formed comprising volunteers aged 18–70 years without occupational lead exposure declaring no shooting activity and any (including administrative) work at shooting ranges, all of whom were residents in central Poland. The number of participants in the control group was 43 (16 women and 27 men). The group profiles are presented in Table 2. All participants completed a questionnaire about age, type of work, personal habits (smoking, alcohol consumption and hobby activities) and use of pharmaceutical products.

**Table 2:** Basic characteristics of the study participants.

Variables	Control group (n = 43)	Study group (n = 45)
Sex n (%)		
Men	27 (62.8)	42 (93.3)
Women	16 (37.2)	3 (6.7)
Age (mean ± SD)	40.4 ± 17.8	42.6 ± 11.5
Education n (%)		
Primary	1 (2.3)	0 (0.0)
Vocational	7 (16.3)	0 (0.0)
Secondary	24 (55.8)	18 (40.0)
Higher	11 (25.6)	27 (60.0)
Current smoking n (%)		
Yes	9 (20.9)	10 (22.2)
No	34 (79.1)	35 (77.8)
Alcohol consumption n (%)		
1—None or < 1 drink/week	14 (32.6)	27 (60.0)
2—1–3 drinks/week	29 (67.4)	18 (40.0)
3—Every day	0 (0.0)	0 (0.0)

### Biological samples

Blood samples were collected using disposable heparinized syringes (LH-Metall-Analytik; Sarstedt Monovette, Numbrecht, Germany) designed for elemental analysis. The activity of 4-aminolevulinate dehydratase (ALA-D) was determined up to 2 h after blood collection. The remaining blood samples from the same syringes were stored frozen at − 80 °C until lead analysis.

First-morning urine samples were collected in containers that had been previously decontaminated (in a 10% HNO_3_ bath for at least 24 h and triple rinsed in deionised water afterwards). Shortly after collection, creatinine concentration was determined, and urine samples were stored frozen at − 80 °C until lead analysis.

Lead concentrations in blood and urine were determined using graphite furnace atomic absorption spectrometry (GF-AAS; HITACHI z8270; Hitachi, Tokyo, Japan) after prior mineralization in a microwave digestive system (CEM MarsExpress; CEM, Matthews, USA) with nitric acid. The limits of detection and precision of the used method were 5.4 μg L^−1^ and 6.2%, respectively.

In addition, intra-laboratory quality control was performed using the following reference materials: “Seronorm-Trace Elements Whole blood level-1” freeze-dried whole blood (Sero, Norway), containing 10 µg L^−1^ of lead; “ClinChek-Control Level-I” freeze-dried urine (RECIPE, Germany), containing 25 µg L^−1^ of lead. The RSD values obtained for the reference material assays were 4.44% for blood and 7.98% for urine (certified and measured values for lead in the reference materials are presented in Table S1 in Supplementary information).

ALA-D activity was measured using a European standardized method with spectrophotometric determination carried out at 555 nm on Hitachi U-2900 spectrophotometer (Hitachi, Tokyo, Japan)^42^. Urine creatinine concentration was determined with the Biolabo creatinine test (kinetic method; Biolabo, Maizy, France) with an use of Hitachi U-2900 spectrophotometer (Hitachi, Tokyo, Japan).

### Personal air samples

Air samples were collected using personal dosimetry in the instructors’ breathing zone, continuously throughout the workday, according to Polish and European standards PN-Z-04008–7:2002/Az1:2004, PN-EN 689:2018 and PN-EN 482:2021-08^43–45^. An individual dust meter (Personal Air Samples; Gilian GilAir3, Sensidyne, St. Petersburg, USA) was used for sampling; the device collects the inhalable fraction of dust at a flow rate of 2 L min^−1^ onto cellulose nitrate membrane filters (pore diameter of 0.8 µm and filter diameter of 25 mm).

In selected places of the shooting range, stationary samples were collected in the same way in order to assess lead concentrations in these areas.

The collected dust was digested with 3 mL of concentrated nitric acid and then diluted to 10 mL with 1% HNO_3_ solution, and the lead concentrations were measured with graphite furnace atomic absorption spectrometry (GF-AAS; HITACHI z8270; Hitachi, Tokyo, Japan).

### Statistical analysis

Microsoft Excel 365 and Statistica 13.1 were used for database management and statistical analysis. The level of significance was set at p < 0.05 (two-sided). Results are shown as mean ± standard deviation (SD).

The qqnorm plot was used to visually assess the normality of distribution and Kolmogorov–Smirnov test for checking the normality of obtained results. As the data were not normally distributed, the results were compared using nonparametric tests. In the cross-sectional analysis, data from all groups were compared using the Kruskal–Wallis test, followed by Dunn’s test.

Biomonitoring data variables were logarithmically transformed to normalize the asymmetric distributions, and relationships between them were assessed using Pearson correlation coefficient. All r values given refer to normalized data, while absolute values are shown in the figures.

### Ethics approval and consent to participate

The study was conducted in accordance with the Declaration of Helsinki and was approved by the Bioethical Committee of Medical University of Lodz, Poland (resolution RNN/86/22/KE of 10 May 2022). All participants were informed about the design and purpose of the study and were free to participate. Confidentiality was guaranteed. Written informed consent was obtained from every participant.

### Supplementary Information

Supplementary Information.

## Data Availability

All data generated or analysed during this study are included in this published article.
