# Supplementary material for: Assessment of lead exposure in indoor shooters in central Poland
Source: Sci Rep. 2023 Aug 3;13:12605. doi: 10.1038/s41598-023-39847-3 (PMC10400594; doi:10.1038/s41598-023-39847-3)
Supplement: Supplementary file 1 — Supplementary Information. [file 41598_2023_39847_MOESM1_ESM.pdf]

SUPPLEMENTARY INFORMATION

Assessment of Lead Exposure in Indoor Shooters in Central Poland

Adam Darago[1], Michał Klimczak[1*], Joanna Jurewicz[2], Małgorzata Kucharska[3] and Anna Kilanowicz[1]

[1] Department of Toxicology, Medical University of Lodz, Muszyńskiego 1, 90-151 Lodz, Poland; adam.darago@umed.lodz.pl; anna.kilanowicz@umed.lodz.pl

[2] Department of Chemical Safety, Nofer Institute of Occupational Medicine, Św. Teresy 8, 91-348 Lodz, Poland; joanna.jurewicz@imp.lodz.pl

[3] Central Laboratory, Nofer Institute of Occupational Medicine, Św. Teresy 8, 91-348 Lodz, Poland; malgorzata.kucharska@imp.lodz.pl

*Correspondence: michal.klimczak@umed.lodz.pl

Table S1. Certified and measured values for lead in the reference materials.

Figure S1. Relationships between BLL and time spent at shooting ranges in study subgroups: (a) < 10 hours per week; (b) 11-20 hours per week and (c) > 20 hours per week.

Figure S2. Relationships between ULL and time spent at shooting ranges in study subgroups: (a) < 10 hours per week; (b) 11-20 hours per week and (c) > 20 hours per week.

Figure S3. Relationships between ALA-D activity and time spent at shooting ranges in study subgroups: (a) < 10 hours per week; (b) 11-20 hours per week and (c) > 20 hours per week.

Figure S4. Relationships between BLL and ULL in study subgroups: (a) < 10 hours per week; (b) 11-20 hours per week and (c) > 20 hours per week.

Figure S5. Relationships between BLL and ALA-D activity in study subgroups: (a) < 10 hours per week; (b) 11-20 hours per week and (c) > 20 hours per week.

Table S1. Certified and measured values for lead in the reference materials.

Reference material	Certified analytical value [$\mu g\ L^{-1}$]	Range [$\mu g\ L^{-1}$]	Measured values [$\mu g\ L^{-1}$]	Accuracy
Seronorm-Trace Elements Whole blood level-1 freeze-dried whole blood (Sero, Norway)	10	7.9-12	10.44±1.08	104.44%
ClinChek-Control Level-I freeze-dried urine (RECIPE, Germany)	25	20-30	26.99±2.67	107.98%

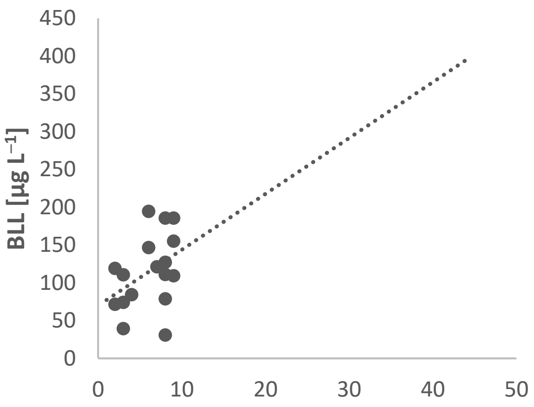

(**a**)

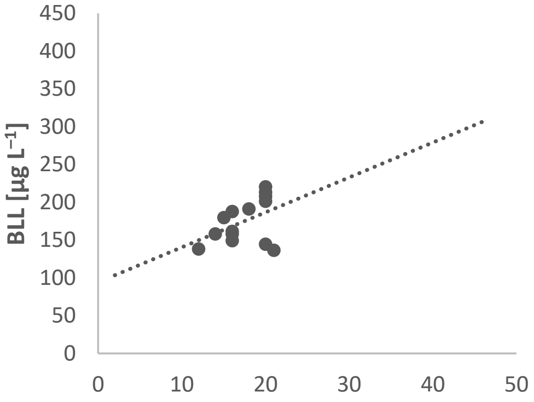

(**b**)

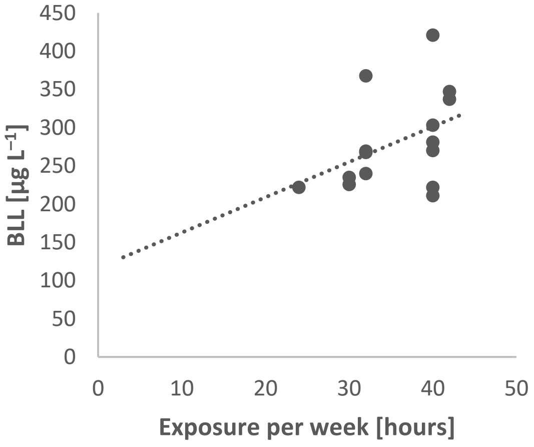

(**c**)

Figure S1. Relationships between BLL and time spent at shooting ranges in study subgroups: (a) < 10 hours per week; (b) 11-20 hours per week and (c) > 20 hours per week.

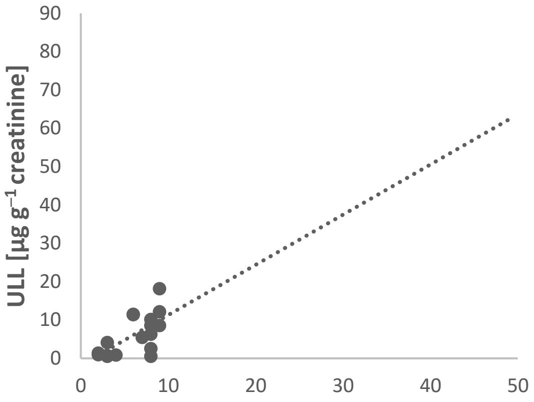

(**a**)

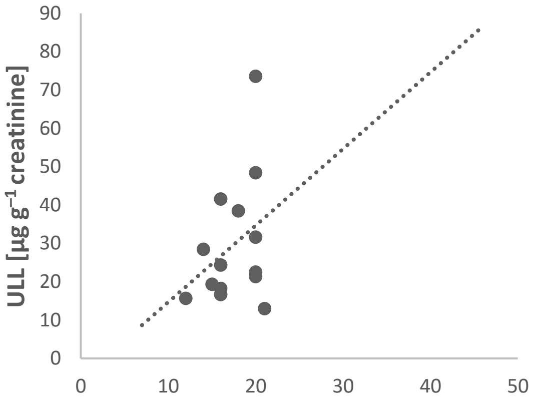

(**b**)

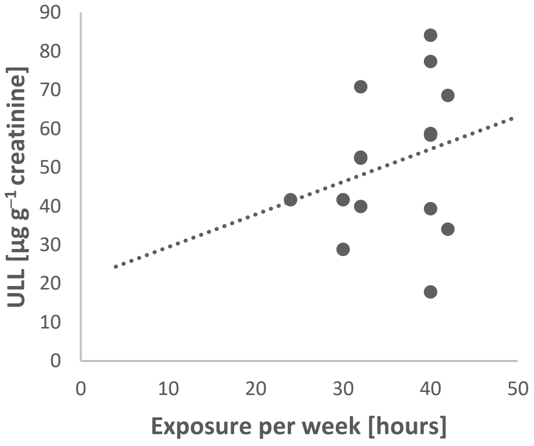

(**c**)

Figure S2. Relationships between ULL and time spent at shooting ranges in study subgroups: (a) < 10 hours per week; (b) 11-20 hours per week and (c) > 20 hours per week.

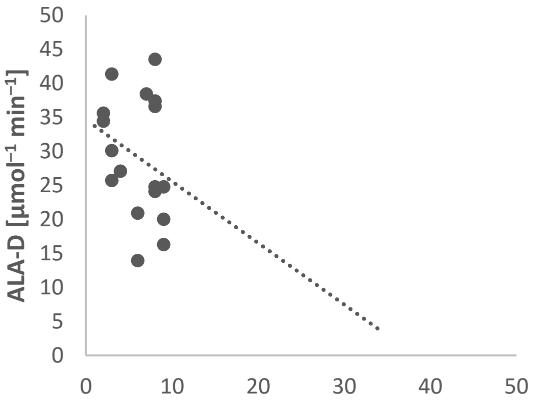

(**a**)

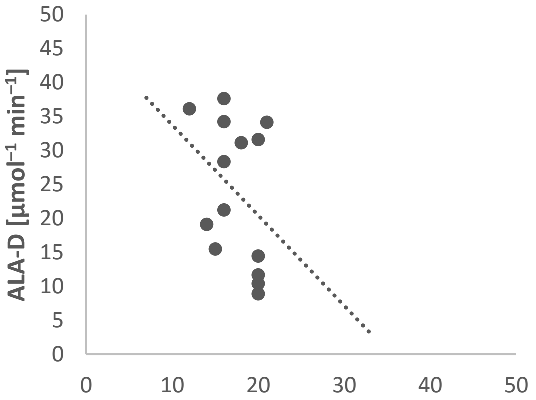

(**b**)

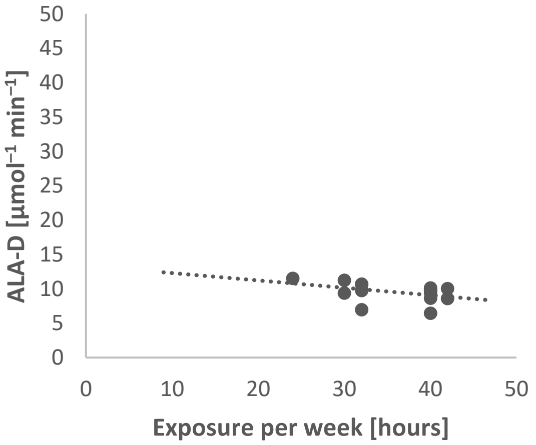

(**c**)

Figure S3. Relationships between ALA-D activity and time spent at shooting ranges in study subgroups: (a) < 10 hours per week; (b) 11-20 hours per week and (c) > 20 hours per week.

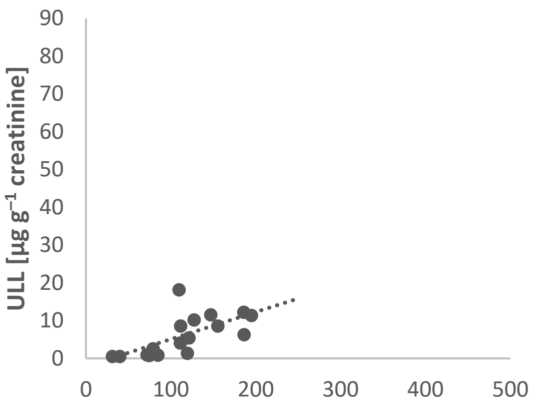

(**a**)

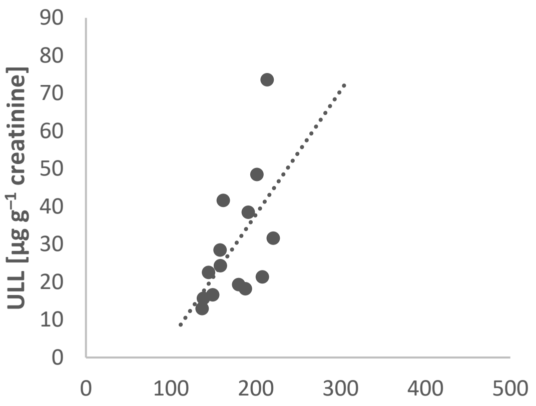

(**b**)

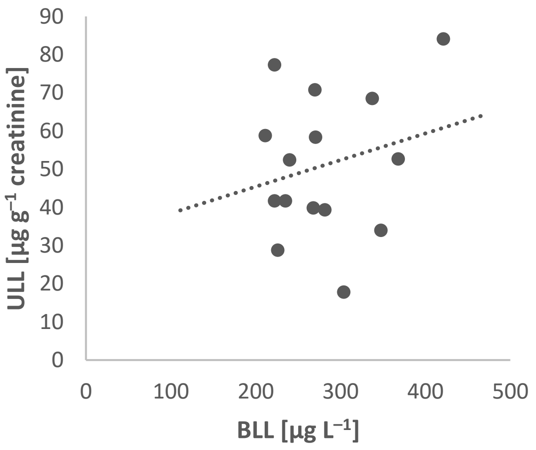

(**c**)

Figure S4. Relationships between BLL and ULL in study subgroups: (a) < 10 hours per week; (b) 11-20 hours per week and (c) > 20 hours per week.

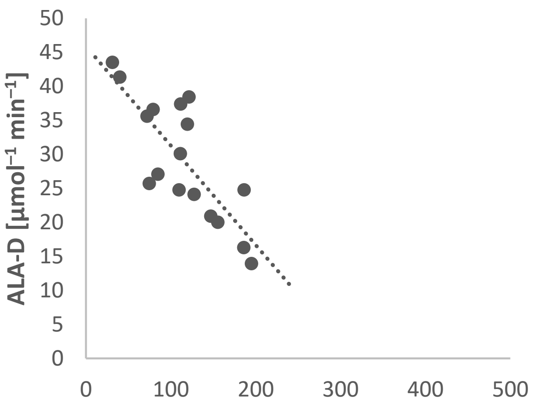

(a)

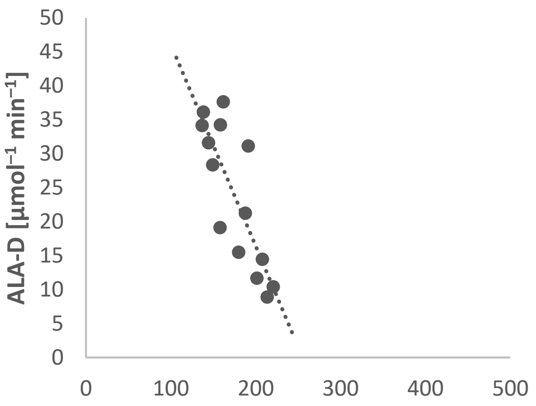

(b)

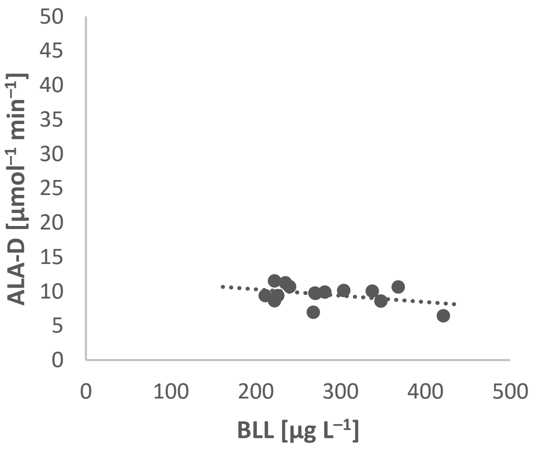

(c)

Figure S5. Relationships between BLL and ALA-D activity in study subgroups: (a) < 10 hours per week; (b) 11-20 hours per week and (c) > 20 hours per week.
